# Poly[[{μ_2_-3-[(1*H*-benzimidazol-1-yl)meth­yl]benzoato}cadmium(II)] 0.1-hydrate]

**DOI:** 10.1107/S1600536810013292

**Published:** 2010-04-17

**Authors:** Li Duan, Xiang-Wen Wu, Jian-Ping Ma

**Affiliations:** a College of Chemistry, Chemical Engineering and Materials Science, Shandong Normal University, Jinan 250014, People’s Republic of China; b State Key Laboratory of Crystal Materials, Shandong University, Jinan 250100, People’s Republic of China

## Abstract

In the title polymeric compound, {[Cd(C_15_H_11_N_2_O_2_)_2_]·0.1H_2_O}_*n*_, the Cd^II^ atom is coordinated by four carboxyl­ate O atoms and two benzimidazole N atoms from four benzimidazolylmethyl­benzoate anions in a distorted octa­hedral geometry. Each anion bridges two Cd atoms through the terminal carboxyl­ate group and an imidazole N atom, forming polymeric complex chains running along the *b* axis. The uncoordinated water mol­ecule is equally disordered over two sites; occupancies were fixed as 0.5 for each disordered component. Weak inter­molecular C—H⋯O hydrogen bonding is present in the crystal structure.

## Related literature

For the use of benzimidazoles and benzimidazole derivatives in the construction of metal-organic frameworks, see: Li *et al.* (2010 ▶); Vijayan *et al.* (2006 ▶).
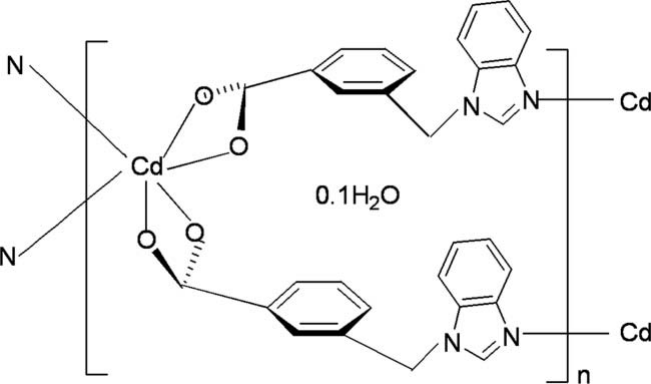


## Experimental

### 

#### Crystal data

[Cd(C_15_H_11_N_2_O_2_)_2_]·0.1H_2_O
*M* *_r_* = 616.72
Monoclinic, 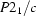

*a* = 12.7770 (18) Å
*b* = 10.8304 (15) Å
*c* = 18.522 (3) Å
β = 95.007 (2)°
*V* = 2553.4 (6) Å^3^
*Z* = 4
Mo *K*α radiation
μ = 0.90 mm^−1^
*T* = 298 K
0.51 × 0.30 × 0.24 mm

#### Data collection

Bruker SMART CCD area-detector diffractometer
Absorption correction: multi-scan (*SADABS*; Sheldrick, 1996 ▶) *T*_min_ = 0.656, *T*_max_ = 0.813
13062 measured reflections
4754 independent reflections
4031 reflections with *I* > 2σ(*I*)
*R*_int_ = 0.025

#### Refinement

*R*[*F* ^2^ > 2σ(*F* ^2^)] = 0.028
*wR*(*F* ^2^) = 0.075
*S* = 1.03
4754 reflections
370 parameters
7 restraints
H-atom parameters constrained
Δρ_max_ = 0.63 e Å^−3^
Δρ_min_ = −0.37 e Å^−3^

### 

Data collection: *SMART* (Bruker, 2000 ▶); cell refinement: *SAINT* (Bruker, 2000 ▶); data reduction: *SAINT*; program(s) used to solve structure: *SHELXTL* (Sheldrick, 2008 ▶); program(s) used to refine structure: *SHELXTL*; molecular graphics: *SHELXTL*; software used to prepare material for publication: *SHELXTL*.

## Supplementary Material

Crystal structure: contains datablocks global, I. DOI: 10.1107/S1600536810013292/xu2743sup1.cif

Structure factors: contains datablocks I. DOI: 10.1107/S1600536810013292/xu2743Isup2.hkl

Additional supplementary materials:  crystallographic information; 3D view; checkCIF report

## Figures and Tables

**Table 1:** Selected bond lengths (Å)

Cd1—O1	2.3559 (17)
Cd1—O2	2.3669 (17)
Cd1—O3	2.4209 (19)
Cd1—O4	2.3063 (19)
Cd1—N1^i^	2.2801 (19)
Cd1—N4^ii^	2.283 (2)

Symmetry codes: (i) 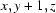; (ii) 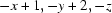.

**Table 2:** Hydrogen-bond geometry (Å, °)

*D*—H⋯*A*	*D*—H	H⋯*A*	*D*⋯*A*	*D*—H⋯*A*
C11—H11⋯O1^iii^	0.93	2.59	3.357 (3)	140
C14—H14⋯O4^iv^	0.93	2.41	3.261 (3)	152
C23—H23*B*⋯O2^v^	0.97	2.58	3.277 (3)	129
C29—H29⋯O3^v^	0.93	2.57	3.424 (3)	154

Symmetry codes: (iii) 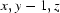; (iv) 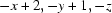; (v) 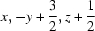.

**Fig. 1.**
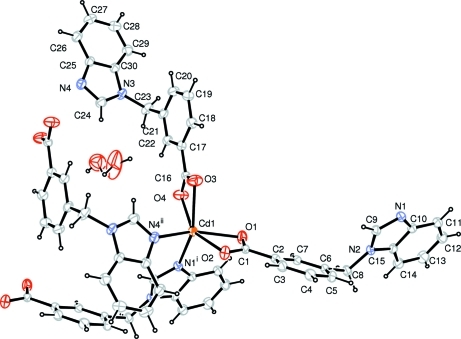
The coordination environment around the Cd atom, displacement ellipsoids drawn at 30% probability level [symmetry codes: (i) x, 1+y, z; (ii) 1-x, 2-y, -z].

**Fig. 2.**
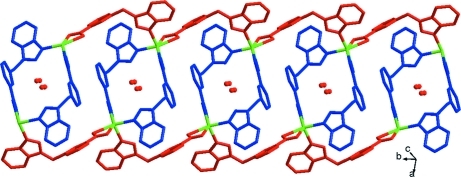
One-dimensional chain structure of (I), disordered water molecules located in the bimetallic ring.

## supplementary crystallographic information

### Comment

The rational design and synthesis of supramolecular complexes are of great interest not only because of their potential applications but also owing to their intriguing structures. Benzimidazole and benzimidazole-containing derivatives acted as one of the useful classes of organic building blocks to construct metal-organic frameworks (MOFs) (Li *et al.*, 2010; Vijayan *et al.*, 2006). Supramolecular complexes based on bent unsymmetric ligands containing benzimidazole and carboxylic acid groups have been less extensively studied, so these bent unsymmetric ligands offer great potential for creating novel frameworks. In the present work, the new bent organic ligand 3-[(1*H*-benzimidazole-1-yl)methyl]benzoic acid (H*L*) was employed in a self-assembly reaction with cadmium (II) iodide under hydrothermal conditions to create the novel supramolecular complex [Cd(C_15_H_11_N_2_O_2_)_2_0.10(H_2_O)]_n_ (I).

The compound structure of (I) is shown in Fig. 1. The asymmetric unit contains two *L* ligands, one Cd(II) and 0.10 water molecule. Compound (I) crystallizes with one unique six-coordinated Cd(II) center in a distorted octahedral {Cd N_2_O_4_} environment involving four O atoms from the carboxylate groups of two *L* ligands and two N atoms from benzimidazole of two other *L* ligands.

Neighboring Cd(II) ions are bound together by the carboxylate groups and terminal benzimidazole N donors of two *L* ligands to form a {Cd_2_L_2_} bimetallic ring in which the diagonal Cd···Cd separation is 9.907 (6) Å. The dihedral angle between benzimidazole ring and benzene ring is 87.618 (113)°. Small amounts of disordered water molecules are located in the bimetallic ring. Each Cd (II) center of the bimetallic ring is further bonded with two other bridging ligands resulting in a novel infinite one-dimensional extended chains structure in the crystallographic *c* axis. The Cd···Cd distance between adjacent bimetallic rings is 10.830 (2) Å, and the dihedral angle of benzimidazole ring and benzene ring of the ligand is 62.467 (82)°, it is worthy noted that the Cd···Cd distance is longer than that of in the bimetallic ring, which may be caused by the different features of the ligand. (Fig. 2).

In the solid state, when viewed down the crystallographic *b* axis, these one-dimensional chains are arranged in an ···AA··· fashion stack through interchain π-π interaction between two benzimidazole ring, the centroid-to-centroid distance of ca. 3.7 Å (Fig. 3).

### Experimental

A mixture of 3-[(1*H*-benzimidazole-1-yl)methyl)methyl]benzoic acid (25.2 mg, 0.10 mmol), CdI_2_ (12.7 mg, 0.10 mmol) and deionized water (2 ml) was sealed in a 5 ml Teflon-lined stainless steel reactor and heated at 453 K for 40 h, and then cooled slowly to room temperature over a period of 50 h. Colorless single crystals were obtained from the reaction mixture.

### Refinement

The lattice water is disordered over two sites, site occupancy factors for each components were refined and converged to 0.048 and 0.046, respectively; in the final cycles of refinement they were fixed as 0.5 for each. H atoms of water molecules were placed at calculated positions and refined with distance constraint of O—H = 0.85±0.001 Å, and U_iso_(H) = 1.5U_eq_(O). Other H atoms were placed in geometrically idealized positions and refined as riding atoms with C—H = 0.93 (aromatic) and 0.97 Å (methylene), U_iso_(H) = 1.2U_eq_(C).

### Crystal data

**Table:**

[Cd(C_15_H_11_N_2_O_2_)_2_]·0.1H_2_O	*F*(000) = 1244
*M**_r_* = 616.72	*D*_x_ = 1.604 Mg m^−^^3^
Monoclinic, *P*2_1_/*c*	Mo *K*α radiation, λ = 0.71073 Å
Hall symbol: -P 2ybc	Cell parameters from 7057 reflections
*a* = 12.7770 (18) Å	θ = 2.2–28.2°
*b* = 10.8304 (15) Å	µ = 0.90 mm^−^^1^
*c* = 18.522 (3) Å	*T* = 298 K
β = 95.007 (2)°	Block, colourless
*V* = 2553.4 (6) Å^3^	0.51 × 0.30 × 0.24 mm
*Z* = 4	

### Data collection

**Table:**

Bruker SMART CCD area-detector diffractometer	4754 independent reflections
Radiation source: fine-focus sealed tube	4031 reflections with *I* > 2σ(*I*)
graphite	*R*_int_ = 0.025
φ and ω scans	θ_max_ = 25.5°, θ_min_ = 2.2°
Absorption correction: multi-scan (*SADABS*; Sheldrick, 1996)	*h* = −15→12
*T*_min_ = 0.656, *T*_max_ = 0.813	*k* = −13→13
13062 measured reflections	*l* = −22→18

### Refinement

**Table:**

Refinement on *F*^2^	Primary atom site location: structure-invariant direct methods
Least-squares matrix: full	Secondary atom site location: difference Fourier map
*R*[*F*^2^ > 2σ(*F*^2^)] = 0.028	Hydrogen site location: inferred from neighbouring sites
*wR*(*F*^2^) = 0.075	H-atom parameters constrained
*S* = 1.03	*w* = 1/[σ^2^(*F*_o_^2^) + (0.0389*P*)^2^ + 0.9163*P*] where *P* = (*F*_o_^2^ + 2*F*_c_^2^)/3
4754 reflections	(Δ/σ)_max_ = 0.002
370 parameters	Δρ_max_ = 0.63 e Å^−^^3^
7 restraints	Δρ_min_ = −0.37 e Å^−^^3^

### Special details

**Table:**

Geometry. All esds (except the esd in the dihedral angle between two l.s. planes) are estimated using the full covariance matrix. The cell esds are taken into account individually in the estimation of esds in distances, angles and torsion angles; correlations between esds in cell parameters are only used when they are defined by crystal symmetry. An approximate (isotropic) treatment of cell esds is used for estimating esds involving l.s. planes.
Refinement. Refinement of *F*^2^ against ALL reflections. The weighted *R*-factor wR and goodness of fit *S* are based on *F*^2^, conventional *R*-factors *R* are based on *F*, with *F* set to zero for negative *F*^2^. The threshold expression of *F*^2^ > σ(*F*^2^) is used only for calculating *R*-factors(gt) etc. and is not relevant to the choice of reflections for refinement. *R*-factors based on *F*^2^ are statistically about twice as large as those based on *F*, and *R*- factors based on ALL data will be even larger.

### Fractional atomic coordinates and isotropic or equivalent isotropic displacement parameters (Å^2^)

**Table:**

	*x*	*y*	*z*	*U*_iso_*/*U*_eq_	Occ. (<1)
C1	0.83100 (17)	0.53070 (18)	−0.16646 (13)	0.0324 (5)	
C2	0.86797 (18)	0.41005 (19)	−0.19581 (12)	0.0346 (5)	
C3	0.8300 (2)	0.3700 (2)	−0.26403 (13)	0.0435 (6)	
H3	0.7817	0.4180	−0.2921	0.052*	
C4	0.8635 (3)	0.2588 (2)	−0.29070 (15)	0.0490 (7)	
H4	0.8373	0.2319	−0.3364	0.059*	
C5	0.9362 (2)	0.1874 (2)	−0.24938 (14)	0.0454 (6)	
H5	0.9596	0.1136	−0.2679	0.055*	
C6	0.9740 (2)	0.2256 (2)	−0.18067 (14)	0.0377 (5)	
C7	0.93982 (18)	0.33745 (19)	−0.15452 (12)	0.0348 (5)	
H7	0.9655	0.3641	−0.1086	0.042*	
C8	1.0543 (2)	0.1491 (2)	−0.13545 (15)	0.0452 (6)	
H8A	1.1135	0.1331	−0.1635	0.054*	
H8B	1.0799	0.1964	−0.0930	0.054*	
C9	0.91692 (18)	−0.0199 (2)	−0.12756 (13)	0.0389 (5)	
H9	0.8618	0.0210	−0.1536	0.047*	
C10	1.00732 (18)	−0.1589 (2)	−0.06694 (12)	0.0360 (5)	
C11	1.0459 (2)	−0.2654 (2)	−0.03163 (14)	0.0426 (6)	
H11	1.0041	−0.3352	−0.0285	0.051*	
C12	1.1482 (2)	−0.2631 (2)	−0.00163 (15)	0.0484 (7)	
H12	1.1758	−0.3327	0.0225	0.058*	
C13	1.2117 (2)	−0.1596 (3)	−0.00641 (14)	0.0531 (7)	
H13	1.2804	−0.1619	0.0147	0.064*	
C14	1.1756 (2)	−0.0534 (2)	−0.04155 (13)	0.0457 (6)	
H14	1.2179	0.0158	−0.0448	0.055*	
C15	1.07229 (18)	−0.0563 (2)	−0.07173 (12)	0.0363 (5)	
C16	0.6286 (2)	0.73215 (19)	−0.00888 (14)	0.0364 (5)	
C17	0.5579 (2)	0.7302 (2)	0.05285 (15)	0.0410 (6)	
C18	0.4722 (2)	0.6511 (2)	0.05053 (14)	0.0466 (6)	
H18	0.4578	0.5994	0.0108	0.056*	
C19	0.4086 (2)	0.6485 (3)	0.10666 (16)	0.0529 (7)	
H19	0.3518	0.5945	0.1050	0.063*	
C20	0.4286 (2)	0.7260 (3)	0.16554 (17)	0.0509 (7)	
H20	0.3849	0.7244	0.2031	0.061*	
C21	0.51359 (19)	0.8060 (2)	0.16893 (13)	0.0432 (6)	
C22	0.57828 (19)	0.8061 (2)	0.11322 (13)	0.0424 (6)	
H22	0.6366	0.8577	0.1159	0.051*	
C23	0.5332 (2)	0.8948 (3)	0.23165 (14)	0.0527 (7)	
H23A	0.5402	0.8491	0.2769	0.063*	
H23B	0.5983	0.9392	0.2272	0.063*	
C24	0.4122 (2)	1.0601 (2)	0.17843 (14)	0.0486 (6)	
H24	0.4465	1.0692	0.1365	0.058*	
C25	0.30136 (18)	1.0798 (2)	0.25785 (13)	0.0392 (5)	
C26	0.2184 (2)	1.1123 (2)	0.29766 (14)	0.0484 (6)	
H26	0.1676	1.1688	0.2800	0.058*	
C27	0.2144 (2)	1.0575 (3)	0.36398 (15)	0.0555 (7)	
H27	0.1595	1.0771	0.3918	0.067*	
C28	0.2903 (2)	0.9734 (3)	0.39098 (15)	0.0547 (7)	
H28	0.2853	0.9395	0.4367	0.066*	
C29	0.3723 (2)	0.9388 (2)	0.35230 (14)	0.0471 (6)	
H29	0.4227	0.8821	0.3702	0.056*	
C30	0.37558 (18)	0.9936 (2)	0.28477 (12)	0.0379 (5)	
Cd1	0.763197 (14)	0.744630 (14)	−0.112674 (10)	0.03670 (8)	
N1	0.90940 (15)	−0.13374 (17)	−0.10222 (10)	0.0377 (4)	
N2	1.01240 (15)	0.03038 (16)	−0.11170 (10)	0.0366 (4)	
N3	0.44586 (16)	0.98322 (19)	0.23252 (11)	0.0432 (5)	
N4	0.32673 (16)	1.12095 (18)	0.19014 (11)	0.0452 (5)	
O1	0.84916 (15)	0.55239 (15)	−0.10022 (10)	0.0514 (4)	
O2	0.78223 (15)	0.60416 (16)	−0.20854 (10)	0.0521 (4)	
O3	0.60131 (16)	0.6789 (2)	−0.06556 (11)	0.0643 (5)	
O4	0.71415 (17)	0.7877 (2)	0.00157 (11)	0.0640 (6)	
O1W	0.593 (5)	0.141 (6)	0.104 (3)	0.116 (19)	0.05
H1O1	0.6060	0.1156	0.0620	0.174*	0.05
H2O1	0.6303	0.2043	0.1160	0.174*	0.05
O2W	0.540 (8)	0.031 (8)	0.020 (3)	0.19 (5)	0.05
H1O2	0.5317	0.0676	−0.0208	0.283*	0.05
H2O2	0.5297	−0.0465	0.0190	0.283*	0.05

### Atomic displacement parameters (Å^2^)

**Table:**

	*U*^11^	*U*^22^	*U*^33^	*U*^12^	*U*^13^	*U*^23^
C1	0.0276 (11)	0.0245 (11)	0.0458 (13)	−0.0005 (9)	0.0073 (9)	0.0016 (10)
C2	0.0350 (12)	0.0288 (11)	0.0412 (12)	−0.0022 (9)	0.0100 (10)	0.0029 (9)
C3	0.0504 (15)	0.0370 (12)	0.0432 (14)	0.0018 (11)	0.0044 (11)	0.0055 (10)
C4	0.067 (2)	0.0428 (15)	0.0380 (14)	−0.0062 (12)	0.0084 (13)	−0.0037 (10)
C5	0.0568 (16)	0.0309 (12)	0.0505 (15)	0.0000 (11)	0.0158 (12)	−0.0062 (11)
C6	0.0386 (13)	0.0256 (11)	0.0503 (15)	−0.0008 (10)	0.0129 (11)	0.0036 (10)
C7	0.0372 (12)	0.0294 (11)	0.0385 (12)	−0.0044 (9)	0.0083 (10)	0.0007 (9)
C8	0.0419 (14)	0.0282 (11)	0.0662 (17)	−0.0012 (10)	0.0090 (12)	0.0062 (11)
C9	0.0350 (12)	0.0326 (12)	0.0493 (14)	0.0032 (10)	0.0046 (10)	0.0035 (10)
C10	0.0404 (13)	0.0336 (11)	0.0351 (12)	0.0027 (10)	0.0094 (10)	−0.0003 (9)
C11	0.0510 (16)	0.0365 (13)	0.0414 (14)	0.0029 (11)	0.0095 (12)	0.0054 (10)
C12	0.0550 (17)	0.0471 (15)	0.0434 (15)	0.0145 (12)	0.0051 (12)	0.0107 (11)
C13	0.0462 (15)	0.0593 (17)	0.0524 (16)	0.0098 (13)	−0.0042 (12)	0.0031 (13)
C14	0.0447 (14)	0.0426 (13)	0.0497 (14)	−0.0023 (11)	0.0042 (11)	−0.0030 (11)
C15	0.0412 (13)	0.0304 (11)	0.0378 (12)	0.0042 (10)	0.0061 (10)	−0.0023 (9)
C16	0.0384 (13)	0.0269 (11)	0.0452 (14)	−0.0031 (9)	0.0112 (11)	−0.0023 (10)
C17	0.0385 (14)	0.0345 (12)	0.0509 (15)	0.0034 (10)	0.0084 (11)	0.0027 (10)
C18	0.0459 (15)	0.0400 (13)	0.0541 (15)	−0.0020 (11)	0.0059 (12)	−0.0005 (11)
C19	0.0378 (14)	0.0499 (15)	0.0721 (19)	−0.0043 (12)	0.0115 (13)	0.0071 (14)
C20	0.0416 (15)	0.0574 (16)	0.0559 (17)	0.0085 (13)	0.0164 (13)	0.0101 (13)
C21	0.0366 (13)	0.0459 (14)	0.0474 (14)	0.0140 (11)	0.0049 (11)	0.0039 (12)
C22	0.0368 (13)	0.0391 (13)	0.0517 (15)	0.0029 (11)	0.0059 (11)	−0.0013 (11)
C23	0.0418 (15)	0.0668 (18)	0.0492 (15)	0.0196 (13)	0.0020 (11)	−0.0045 (13)
C24	0.0482 (15)	0.0547 (15)	0.0440 (14)	0.0086 (13)	0.0100 (11)	0.0013 (12)
C25	0.0355 (12)	0.0361 (12)	0.0461 (13)	0.0013 (10)	0.0033 (10)	−0.0067 (10)
C26	0.0395 (14)	0.0475 (14)	0.0584 (16)	0.0066 (12)	0.0058 (12)	−0.0059 (12)
C27	0.0398 (14)	0.0694 (18)	0.0588 (17)	0.0026 (14)	0.0134 (12)	−0.0082 (14)
C28	0.0491 (16)	0.0676 (19)	0.0482 (15)	−0.0075 (14)	0.0084 (12)	0.0032 (13)
C29	0.0426 (14)	0.0488 (14)	0.0492 (15)	0.0010 (12)	0.0001 (11)	0.0005 (12)
C30	0.0337 (12)	0.0384 (12)	0.0413 (13)	0.0008 (10)	0.0016 (10)	−0.0059 (10)
Cd1	0.03766 (12)	0.02956 (11)	0.04360 (12)	0.00121 (7)	0.00763 (8)	−0.00008 (7)
N1	0.0387 (11)	0.0303 (10)	0.0445 (11)	−0.0010 (8)	0.0060 (8)	0.0040 (8)
N2	0.0372 (11)	0.0261 (9)	0.0472 (11)	0.0011 (8)	0.0074 (8)	0.0017 (8)
N3	0.0388 (11)	0.0478 (12)	0.0430 (11)	0.0139 (9)	0.0037 (9)	−0.0014 (9)
N4	0.0440 (12)	0.0424 (11)	0.0493 (12)	0.0095 (10)	0.0047 (9)	0.0013 (9)
O1	0.0683 (13)	0.0342 (9)	0.0515 (11)	0.0076 (9)	0.0046 (9)	−0.0052 (8)
O2	0.0575 (11)	0.0395 (9)	0.0585 (11)	0.0113 (9)	0.0012 (9)	−0.0032 (8)
O3	0.0602 (13)	0.0797 (14)	0.0554 (12)	−0.0188 (11)	0.0183 (10)	−0.0185 (11)
O4	0.0643 (14)	0.0727 (12)	0.0587 (13)	−0.0255 (11)	0.0259 (10)	−0.0174 (11)
O1W	0.11 (2)	0.11 (2)	0.12 (2)	−0.003 (10)	0.007 (10)	0.002 (10)
O2W	0.30 (13)	0.18 (8)	0.08 (6)	0.15 (9)	−0.04 (5)	0.03 (5)

### Geometric parameters (Å, °)

**Table:**

Cd1—O1	2.3559 (17)	C15—N2	1.384 (3)
Cd1—O2	2.3669 (17)	C16—O3	1.221 (3)
Cd1—O3	2.4209 (19)	C16—O4	1.248 (3)
Cd1—O4	2.3063 (19)	C16—C17	1.518 (3)
Cd1—N1^i^	2.2801 (19)	C17—C18	1.388 (3)
Cd1—N4^ii^	2.283 (2)	C17—C22	1.394 (4)
C1—O2	1.243 (3)	C18—C19	1.375 (4)
C1—O1	1.251 (3)	C18—H18	0.9300
C1—C2	1.507 (3)	C19—C20	1.382 (4)
C2—C3	1.383 (3)	C19—H19	0.9300
C2—C7	1.387 (3)	C20—C21	1.386 (4)
C3—C4	1.384 (3)	C20—H20	0.9300
C3—H3	0.9300	C21—C22	1.377 (3)
C4—C5	1.386 (4)	C21—C23	1.512 (4)
C4—H4	0.9300	C22—H22	0.9300
C5—C6	1.385 (4)	C23—N3	1.472 (3)
C5—H5	0.9300	C23—H23A	0.9700
C6—C7	1.390 (3)	C23—H23B	0.9700
C6—C8	1.513 (4)	C24—N4	1.310 (3)
C7—H7	0.9300	C24—N3	1.344 (3)
C8—N2	1.475 (3)	C24—H24	0.9300
C8—H8A	0.9700	C25—C26	1.389 (3)
C8—H8B	0.9700	C25—C30	1.392 (3)
C9—N1	1.326 (3)	C25—N4	1.395 (3)
C9—N2	1.345 (3)	C26—C27	1.369 (4)
C9—H9	0.9300	C26—H26	0.9300
C10—N1	1.387 (3)	C27—C28	1.392 (4)
C10—C15	1.395 (3)	C27—H27	0.9300
C10—C11	1.395 (3)	C28—C29	1.372 (4)
C11—C12	1.376 (4)	C28—H28	0.9300
C11—H11	0.9300	C29—C30	1.389 (3)
C12—C13	1.391 (4)	C29—H29	0.9300
C12—H12	0.9300	C30—N3	1.381 (3)
C13—C14	1.380 (4)	O1W—H1O1	0.8500
C13—H13	0.9300	O1W—H2O1	0.8501
C14—C15	1.388 (3)	O2W—H1O2	0.8499
C14—H14	0.9300	O2W—H2O2	0.8500
			
O2—C1—O1	122.3 (2)	C21—C22—H22	119.4
O2—C1—C2	119.0 (2)	C17—C22—H22	119.4
O1—C1—C2	118.7 (2)	N3—C23—C21	110.3 (2)
C3—C2—C7	119.2 (2)	N3—C23—H23A	109.6
C3—C2—C1	120.4 (2)	C21—C23—H23A	109.6
C7—C2—C1	120.4 (2)	N3—C23—H23B	109.6
C2—C3—C4	120.3 (2)	C21—C23—H23B	109.6
C2—C3—H3	119.9	H23A—C23—H23B	108.1
C4—C3—H3	119.9	N4—C24—N3	113.9 (2)
C3—C4—C5	120.1 (3)	N4—C24—H24	123.0
C3—C4—H4	119.9	N3—C24—H24	123.0
C5—C4—H4	119.9	C26—C25—C30	120.5 (2)
C6—C5—C4	120.3 (2)	C26—C25—N4	130.1 (2)
C6—C5—H5	119.8	C30—C25—N4	109.4 (2)
C4—C5—H5	119.8	C27—C26—C25	117.2 (2)
C5—C6—C7	119.0 (2)	C27—C26—H26	121.4
C5—C6—C8	120.8 (2)	C25—C26—H26	121.4
C7—C6—C8	120.2 (2)	C26—C27—C28	121.8 (2)
C2—C7—C6	121.0 (2)	C26—C27—H27	119.1
C2—C7—H7	119.5	C28—C27—H27	119.1
C6—C7—H7	119.5	C29—C28—C27	122.1 (3)
N2—C8—C6	113.3 (2)	C29—C28—H28	119.0
N2—C8—H8A	108.9	C27—C28—H28	119.0
C6—C8—H8A	108.9	C28—C29—C30	116.0 (2)
N2—C8—H8B	108.9	C28—C29—H29	122.0
C6—C8—H8B	108.9	C30—C29—H29	122.0
H8A—C8—H8B	107.7	N3—C30—C29	132.0 (2)
N1—C9—N2	113.1 (2)	N3—C30—C25	105.6 (2)
N1—C9—H9	123.5	C29—C30—C25	122.4 (2)
N2—C9—H9	123.5	N1^i^—Cd1—N4^ii^	92.79 (7)
N1—C10—C15	109.32 (19)	N1^i^—Cd1—O4	95.25 (7)
N1—C10—C11	130.6 (2)	N4^ii^—Cd1—O4	106.60 (8)
C15—C10—C11	120.1 (2)	N1^i^—Cd1—O1	97.51 (7)
C12—C11—C10	117.3 (2)	N4^ii^—Cd1—O1	146.09 (7)
C12—C11—H11	121.3	O4—Cd1—O1	104.51 (8)
C10—C11—H11	121.3	N1^i^—Cd1—O2	107.34 (7)
C11—C12—C13	121.9 (2)	N4^ii^—Cd1—O2	90.99 (7)
C11—C12—H12	119.1	O4—Cd1—O2	150.71 (7)
C13—C12—H12	119.1	O1—Cd1—O2	55.10 (6)
C14—C13—C12	121.9 (3)	N1^i^—Cd1—O3	149.16 (7)
C14—C13—H13	119.0	N4^ii^—Cd1—O3	90.86 (8)
C12—C13—H13	119.0	O4—Cd1—O3	54.52 (7)
C13—C14—C15	116.0 (2)	O1—Cd1—O3	96.36 (7)
C13—C14—H14	122.0	O2—Cd1—O3	103.21 (6)
C15—C14—H14	122.0	C9—N1—C10	105.02 (19)
N2—C15—C14	131.5 (2)	C9—N1—Cd1^iii^	126.26 (16)
N2—C15—C10	105.7 (2)	C10—N1—Cd1^iii^	128.69 (14)
C14—C15—C10	122.9 (2)	C9—N2—C15	106.92 (18)
O3—C16—O4	122.7 (2)	C9—N2—C8	129.3 (2)
O3—C16—C17	119.8 (2)	C15—N2—C8	123.54 (19)
O4—C16—C17	117.4 (2)	C24—N3—C30	106.6 (2)
C18—C17—C22	118.8 (2)	C24—N3—C23	126.0 (2)
C18—C17—C16	120.3 (2)	C30—N3—C23	126.9 (2)
C22—C17—C16	120.9 (2)	C24—N4—C25	104.5 (2)
C19—C18—C17	120.4 (3)	C24—N4—Cd1^ii^	126.74 (17)
C19—C18—H18	119.8	C25—N4—Cd1^ii^	128.66 (15)
C17—C18—H18	119.8	C1—O1—Cd1	91.43 (13)
C18—C19—C20	120.2 (3)	C1—O2—Cd1	91.13 (14)
C18—C19—H19	119.9	C16—O3—Cd1	89.01 (15)
C20—C19—H19	119.9	C16—O4—Cd1	93.73 (15)
C19—C20—C21	120.4 (2)	H1O1—O1W—H2O1	111.1
C19—C20—H20	119.8	O2W^iv^—O2W—H1O1	167.2
C21—C20—H20	119.8	O2W^iv^—O2W—H1O2	73.8
C22—C21—C20	119.1 (2)	H1O1—O2W—H1O2	102.1
C22—C21—C23	120.3 (2)	O2W^iv^—O2W—H2O2	53.3
C20—C21—C23	120.6 (2)	H1O1—O2W—H2O2	136.9
C21—C22—C17	121.2 (2)	H1O2—O2W—H2O2	115.8
			
O2—C1—C2—C3	−13.5 (3)	N2—C9—N1—Cd1^iii^	178.46 (14)
O1—C1—C2—C3	165.4 (2)	C15—C10—N1—C9	0.5 (2)
O2—C1—C2—C7	167.1 (2)	C11—C10—N1—C9	−178.3 (2)
O1—C1—C2—C7	−14.1 (3)	C15—C10—N1—Cd1^iii^	−177.58 (14)
C7—C2—C3—C4	−0.1 (3)	C11—C10—N1—Cd1^iii^	3.7 (3)
C1—C2—C3—C4	−179.6 (2)	N1—C9—N2—C15	−1.0 (3)
C2—C3—C4—C5	−0.6 (4)	N1—C9—N2—C8	174.0 (2)
C3—C4—C5—C6	1.3 (4)	C14—C15—N2—C9	179.9 (2)
C4—C5—C6—C7	−1.4 (4)	C10—C15—N2—C9	1.2 (2)
C4—C5—C6—C8	−179.3 (2)	C14—C15—N2—C8	4.6 (4)
C3—C2—C7—C6	0.0 (3)	C10—C15—N2—C8	−174.1 (2)
C1—C2—C7—C6	179.53 (19)	C6—C8—N2—C9	3.7 (3)
C5—C6—C7—C2	0.7 (3)	C6—C8—N2—C15	177.9 (2)
C8—C6—C7—C2	178.6 (2)	N4—C24—N3—C30	0.4 (3)
C5—C6—C8—N2	−68.0 (3)	N4—C24—N3—C23	173.8 (2)
C7—C6—C8—N2	114.1 (2)	C29—C30—N3—C24	−178.3 (3)
N1—C10—C11—C12	179.6 (2)	C25—C30—N3—C24	−0.2 (3)
C15—C10—C11—C12	0.9 (3)	C29—C30—N3—C23	8.4 (4)
C10—C11—C12—C13	−0.4 (4)	C25—C30—N3—C23	−173.5 (2)
C11—C12—C13—C14	−0.1 (4)	C21—C23—N3—C24	−57.8 (3)
C12—C13—C14—C15	0.0 (4)	C21—C23—N3—C30	114.2 (3)
C13—C14—C15—N2	−178.0 (2)	N3—C24—N4—C25	−0.5 (3)
C13—C14—C15—C10	0.5 (3)	N3—C24—N4—Cd1^ii^	−177.37 (16)
N1—C10—C15—N2	−1.1 (2)	C26—C25—N4—C24	−179.8 (3)
C11—C10—C15—N2	177.8 (2)	C30—C25—N4—C24	0.3 (3)
N1—C10—C15—C14	−179.9 (2)	C26—C25—N4—Cd1^ii^	−2.9 (4)
C11—C10—C15—C14	−1.0 (3)	C30—C25—N4—Cd1^ii^	177.13 (16)
O3—C16—C17—C18	11.5 (4)	O2—C1—O1—Cd1	−0.3 (2)
O4—C16—C17—C18	−167.4 (2)	C2—C1—O1—Cd1	−179.18 (17)
O3—C16—C17—C22	−169.4 (2)	N1^i^—Cd1—O1—C1	−105.75 (14)
O4—C16—C17—C22	11.7 (4)	N4^ii^—Cd1—O1—C1	0.8 (2)
C22—C17—C18—C19	0.4 (4)	O4—Cd1—O1—C1	156.81 (13)
C16—C17—C18—C19	179.5 (2)	O2—Cd1—O1—C1	0.18 (12)
C17—C18—C19—C20	0.8 (4)	O3—Cd1—O1—C1	101.87 (14)
C18—C19—C20—C21	−0.6 (4)	O1—C1—O2—Cd1	0.3 (2)
C19—C20—C21—C22	−0.7 (4)	C2—C1—O2—Cd1	179.18 (17)
C19—C20—C21—C23	177.3 (2)	N1^i^—Cd1—O2—C1	86.91 (14)
C20—C21—C22—C17	2.0 (4)	N4^ii^—Cd1—O2—C1	−179.86 (14)
C23—C21—C22—C17	−176.1 (2)	O4—Cd1—O2—C1	−51.9 (2)
C18—C17—C22—C21	−1.8 (4)	O1—Cd1—O2—C1	−0.19 (12)
C16—C17—C22—C21	179.1 (2)	O3—Cd1—O2—C1	−88.75 (14)
C22—C21—C23—N3	114.7 (3)	O4—C16—O3—Cd1	−1.1 (3)
C20—C21—C23—N3	−63.4 (3)	C17—C16—O3—Cd1	−180.0 (2)
C30—C25—C26—C27	1.0 (4)	N1^i^—Cd1—O3—C16	−12.4 (2)
N4—C25—C26—C27	−178.9 (2)	N4^ii^—Cd1—O3—C16	−109.26 (16)
C25—C26—C27—C28	0.4 (4)	O4—Cd1—O3—C16	0.63 (15)
C26—C27—C28—C29	−1.2 (5)	O1—Cd1—O3—C16	103.93 (16)
C27—C28—C29—C30	0.5 (4)	O2—Cd1—O3—C16	159.52 (15)
C28—C29—C30—N3	178.7 (3)	O3—C16—O4—Cd1	1.2 (3)
C28—C29—C30—C25	0.8 (4)	C17—C16—O4—Cd1	−179.96 (18)
C26—C25—C30—N3	−180.0 (2)	N1^i^—Cd1—O4—C16	172.73 (17)
N4—C25—C30—N3	0.0 (3)	N4^ii^—Cd1—O4—C16	78.24 (18)
C26—C25—C30—C29	−1.6 (4)	O1—Cd1—O4—C16	−88.09 (18)
N4—C25—C30—C29	178.3 (2)	O2—Cd1—O4—C16	−46.4 (3)
N2—C9—N1—C10	0.3 (3)	O3—Cd1—O4—C16	−0.61 (15)

Symmetry codes: (i) *x*, *y*+1, *z*; (ii) −*x*+1, −*y*+2, −*z*; (iii) *x*, *y*−1, *z*; (iv) −*x*+1, −*y*, −*z*.

### Hydrogen-bond geometry (Å, °)

**Table:**

*D*—H···*A*	*D*—H	H···*A*	*D*···*A*	*D*—H···*A*
C11—H11···O1^iii^	0.93	2.59	3.357 (3)	140
C14—H14···O4^v^	0.93	2.41	3.261 (3)	152
C23—H23B···O2^vi^	0.97	2.58	3.277 (3)	129
C29—H29···O3^vi^	0.93	2.57	3.424 (3)	154

Symmetry codes: (iii) *x*, *y*−1, *z*; (v) −*x*+2, −*y*+1, −*z*; (vi) *x*, −*y*+3/2, *z*+1/2.

## References

[bb1] Bruker (2000). *SMART* and *SAINT* Bruker AXS Inc., Madison, Wisconsin, USA.

[bb2] Li, H., Wei, Z., Gong, Q. & Han, Q. (2010). *Acta Cryst.* E**66**, m267. 10.1107/S1600536810002837 PMC2983580 21580218

[bb3] Sheldrick, G. M. (1996). *SADABS* University of Göttingen, Germany.

[bb4] Sheldrick, G. M. (2008). *Acta Cryst.* A**64**, 112–122. 10.1107/S0108767307043930 18156677

[bb5] Vijayan, N., Bhagavannarayana, G., Balamurugan, N., Babu, R. R., Maurya, K. K., Gopalakrishnan, R. & Ramasamy, P. (2006). *J. Cryst. Growth*, **293**, 318–323.

